# *In vivo* detection of cucurbit[6]uril, a hyperpolarized xenon contrast agent for a xenon magnetic resonance imaging biosensor

**DOI:** 10.1038/srep41027

**Published:** 2017-01-20

**Authors:** Francis T. Hane, Tao Li, Peter Smylie, Raiili M. Pellizzari, Jennifer A. Plata, Brenton DeBoef, Mitchell S. Albert

**Affiliations:** 1 Department of Chemistry, Lakehead University, 955 Oliver Rd., Thunder Bay ON P7B 5E1, Canada; 2 Thunder Bay Regional Research institute, 980 Oliver Rd., Thunder Bay ON P7B 5E1, Canada; 3 Department of Chemistry, University of Rhode Island, 140 Flagg Road, Kingston RI 02881, USA; 4 Northern Ontario School of Medicine, 955 Oliver Rd., Thunder Bay ON P7B 5E1, Canada

## Abstract

The Hyperpolarized gas Chemical Exchange Saturation Transfer (HyperCEST) Magnetic Resonance (MR) technique has the potential to increase the sensitivity of a hyperpolarized xenon-129 MRI contrast agent. Signal enhancement is accomplished by selectively depolarizing the xenon within a cage molecule which, upon exchange, reduces the signal in the dissolved phase pool. Herein we demonstrate the *in vivo* detection of the cucurbit[6]uril (CB6) contrast agent within the vasculature of a living rat. Our work may be used as a stepping stone towards using the HyperCEST technique as a molecular imaging modality.

Hyperpolarized (HP) gas Chemical Exchange Saturation Transfer (HyperCEST) magnetic resonance imaging (MRI) contrast agents increase the sensitivity of HP gas MRI by several orders of magnitude^1^. These contrast agents can be conjugated with affinity tags to create xenon MR imaging biosensors to be used as a part of a potential molecular imaging modality^2^,^3^,^4^. Molecular imaging allows for the molecular detection, localization, and characterization of areas of pathology within the body using medical imaging^5^ without the need for invasive surgical biopsies. A number of imaging modalities have been demonstrated to be adept at molecular imaging including magnetic resonance imaging (MRI)^6^, positron emission tomography (PET)^7^, and single positron computed tomography (SPECT)^8^; each are limited by their own specific characteristics. These imaging modalities rely on an injectable molecular probe which combines a detectable molecule and an affinity tag or antibody that binds to the molecule of interest within the body. Currently, the most common molecular imaging method is fluoro-deoxyglucose PET (FDG-PET). In FDG-PET, a radioactive ^18^F isotope is substituted for a hydroxyl group within deoxyglucose to create FDG and is used to detect areas of the body with abnormally high glucose metabolism which is an indicator of tumour neogenesis^9^. In a recent example of molecular imaging, Viola *et al*. used an iron nanoparticle displaying magnetic resonance, conjugated with an anti-amyloid oligomer antibody to detect and predict the onset of Alzheimer’s disease in rodents^10^. Hyperpolarized (HP) xenon MRI^11^ biosensors have the potential to characterize areas of pathologies within the body^2^. Hyperpolarization creates a nuclear spin polarization of xenon far beyond thermal equilibrium conditions, allowing a sensitivity increase of up to 100,000 times above thermally polarized nuclei^11^. The basis of HyperCEST imaging biosensors is a cage molecule, which, when interacting with a HP xenon atom, acts as a HP xenon MRI contrast agent. The cage molecule is a supramolecular host that can reversibly encapsulate a guest HP xenon atom. The encapsulation and diffusion of ^129^Xe in and out of the cage molecule provides a unique and detectable magnetic resonance (MR) chemical shift frequency for the xenon atom inside the cage^1^,^3^,^12^,^13^,^14^,^15^,^16^. The HyperCEST MRI technique multiplies the signal enhancement of the hyperpolarization of ^129^Xe^17^ with the chemical exchange signal enhancement produced by the CEST MRI pulse sequence to provide a signal enhancement of up to one billion times above thermally polarized conditions^1^. To provide contrast, a HyperCEST saturation pre-pulse is applied at the chemical shift frequency of xenon within the cage molecule, thereby depolarizing the xenon atoms encapsulated by the cage. As the depolarized xenon atoms exchange out of the cage molecule, they are replaced by polarized atoms from the reservoir of dissolved ^129^Xe atoms in solution. Because of this diffusion, one Xe host can affect the magnetization of many Xe atoms causing a decrease of polarized nuclei in the dissolved-phase xenon reservoir, thereby reducing its signal intensity^1^. Similarly, in HyperCEST MR imaging, by comparing the “saturation” on-resonance xenon image with the ”control” off-resonance image, a HyperCEST saturation map can be created, showing the location of the MR active molecules^18^. By conjugating the cage molecule with an affinity tag, a HP xenon MR imaging biosensor can be created that can potentially detect spatially localized areas of pathology within the body^18^,^19^,^20^,^21^. A variety of xenon encapsulating molecules and structures have been proposed and tested *in vitro* including cryptophanes^13^,^19^,^20^,^21^,^22^,^23^,^24^, cucurbiturils^14^,^25^,^26^, gas vesicles^18^, lipid structures^27^,^28^ as well as others^13^,^29^. While cryptophanes have been the most popular xenon encapsulating molecules in the past, cucurbiturils have recently gained popularity in light of their superior exchange rates and HyperCEST characteristics^26^,^30^. Other similar methods of signal enhancement that rely upon chemical exchange, such as xenon transfer contrast (XTC), have also been reported^31^.

The HyperCEST technique provides high resolution MR images with a theoretical sensitivity comparable to Positron Emission Tomography (PET), but with the spatial resolution of MRI. Despite this promise, xenon MR imaging biosensors have yet to be detected within a living animal model following their intravenous administration. In this report, we demonstrate what we believe to be the first example of the *in vivo* detection of cucurbit[6]uril (CB6), a HP Xe gas MRI contrast agent, using HyperCEST-enhanced ^129^Xe MRI, within the vasculature of a living rat. This MR active contrast agent could potentially be conjugated with an affinity tag to create such a HP xenon MR imaging biosensor. By having the rat breathe xenon gas, which dissolves in the blood and interacts with the injected CB6 cages circulating in the vasculature, we were able to successfully detect the presence of CB6 in the brain, heart, liver, aorta, and the kidneys. These results are a stepping stone needed to translate this technique from *in vitro* studies to more advanced imaging biosensor studies.

## Results

**Figure 1:**
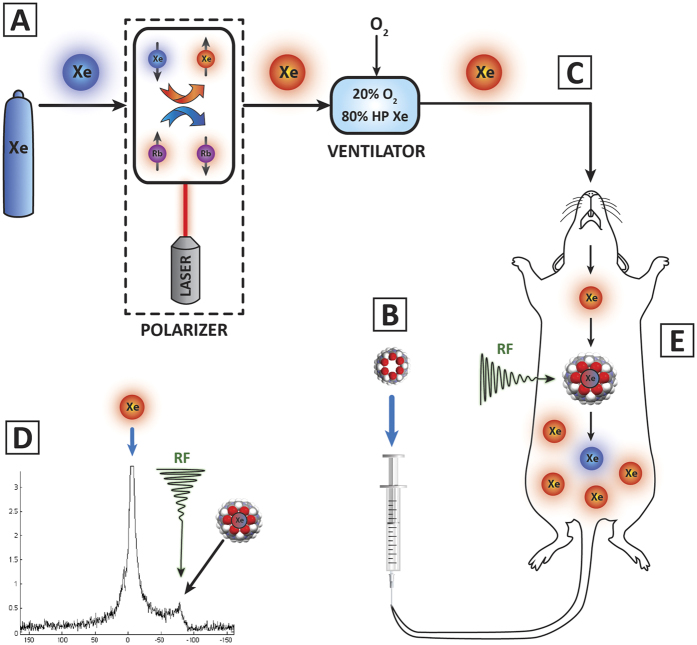
(**A**) Xenon is polarized using Spin Exchange Optical Pumping (SEOP). In SEOP, xenon flows into a chamber containing rubidium gas vapor in the presence of a magnetic field. The rubidium is excited by a laser. Xe colliding with the Rb results in a spin exchange, whereby a greater proportion of Xe atoms are in the lower energy spin state (red). (**B**) The CB6 solution is intravenously injected into the tail vein and allowed to bio-distribute. (**C**) The rat is mechanically ventilated with a mixture of 80% xenon/20% oxygen. The rat inhales HP Xe gas which enters the vasculature via the lungs. The HP Xe interacts with the CB6 cage, diffusing in and out of the cage. Figure created by Craig Christoff of Cryodragon Inc. (**D**) A HyperCEST saturation pre-pulse is applied at the chemical shift frequency of the Xe-CB6 complex (123.4 ppm), depolarizing the Xe in the cage (blue). Because of the exchange of xenon in and out of the cage molecule there is in a reduction of signal in the dissolved reservoir. (**E**) During acquisition, an RF pulse is applied at the chemical shift of Xe-CB6, depolarizing only the Xe within the CB6 cage. As the depolarized xenon exchanges out of the CB6 cage, it reduces the pool of polarized (detectable) Xe atoms (red). The reduction of MR signal compared to a control signal indicates the presence of CB6 cages.

**Figure 2:**
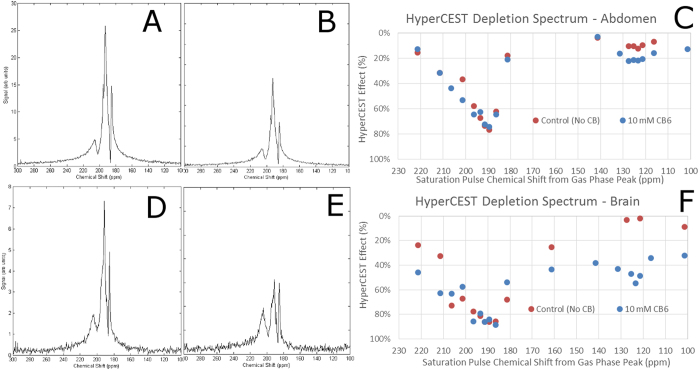
MR spectra and images for the abdomen of a Sprague-Dawley rat following IV injection of 3 mL of 10 mM CB6 solution. (**A**) Free induction decay (FID) spectrum acquired in the abdominal region following the application of an off-resonance (control) pre-pulse at +260 ppm. (**B**) FID spectrum acquired following the application of an on-resonance HyperCEST pulse at +123.4 ppm. Notice the reduction in the SNR of the primary peak indicating a HyperCEST depletion. (**C**) HyperCEST depletion spectrum of the abdomen showing the HyperCEST effect as a function of the chemical shift offset of the saturation pulse. Notice a HyperCEST effect at the CB6 resonance frequency (+123.4 ppm) compared to its absence in the control experiment. (**D**–**F**) Identical to (**A**–**C**) but acquired at the head.

3 mL of 10 mM CB6 solution was injected into the tail vein catheter of a Sprague-Dawley (SD) rat and was allowed to bio-distribute for 30 minutes. An endotracheal tube was surgically placed in the trachea of the anaesthetized rat, and the rat was ventilated with 100% oxygen using a custom made ventilator. The rat was placed into a whole body custom made rat RF coil. Ten seconds prior to xenon MR acquisition, the rat was continuously ventilated with a gas mixture of 80% xenon/20% oxygen (Fig. 1). We acquired magnetic resonance spectra (MRS) (Fig. 2A and D) from both the abdomen and head of the rat. The MR spectrum from the abdomen revealed three resolvable peaks at +184 ppm, +192.5 ppm, and +207 ppm with respect to the ^129^Xe gas phase chemical shift frequency (referenced to 0 ppm) (Fig. 2A). The chemical shift of these peaks approximate those that have been previously assigned to fat, lung parenchyma, and blood respectively^32^, although interspecies differences have been known to occur^32^. We did not observe a peak corresponding to the ^129^Xe-CB6 peak, as previously reported^14^. We attribute this to a variety of susceptibility and line broadening effects occurring *in vivo*. By applying a HyperCEST pre-pulse train at the known chemical shift frequency of ^129^Xe-CB6 (+123.4 ppm)^16^, we acquired an MR spectrum with a 22% reduction in signal intensity compared to the off-resonance control spectrum (Fig. 2C). We repeated this technique after the rat was placed into a custom made rat head RF coil (Fig. 2D–F). By applying a HyperCEST pre-pulse (+123.4 ppm), we observed a 55% reduction in signal intensity of the MR signal from the brain of the rat (Fig. 2F). We observed four xenon peaks at +185 ppm, +191 ppm, +193 ppm, +205 ppm. Based on previously report results^33^,^34^, we assign these peaks to muscle, white matter, grey matter, and red blood cells, respectively.

**Figure 3:**
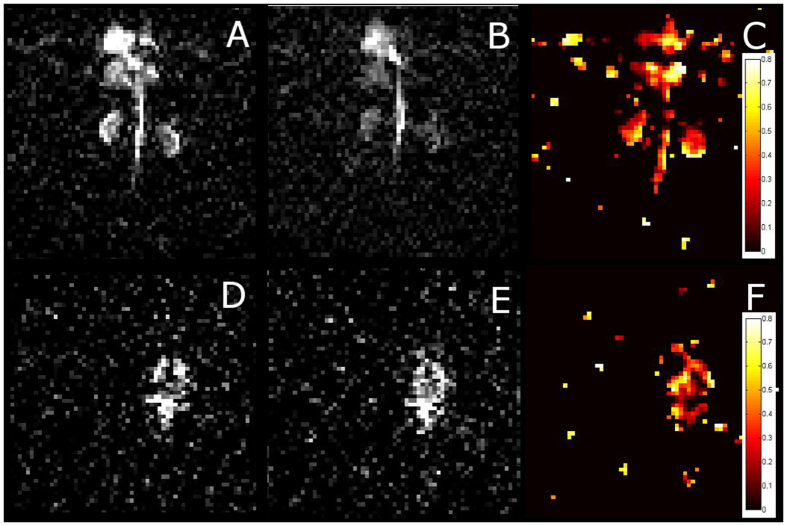
Xe MR images of a Sprague-Dawley rat following IV injection of 3 mL of 10 mM CB6 solution. (**A**) A 2D gradient echo (GE) Xe MR image of the abdomen following the application of an off-resonance pre-pulse (+260 ppm). (**B**) Same as (**A**) but following an on-resonance HyperCEST saturation pre-pulse (+123.4), which destroys the polarization of the Xe in the CB6 cage. As these depolarized Xe atoms leave the cage, they reduce the pool of polarized Xe in the blood, thereby reducing the MR signal. (**C**) A saturation map constructed by subtracting, pixel-by-pixel, the on-resonance HyperCEST image from the off-resonance control image, and dividing by the off-resonance control image. This measures signal depletion and indicates the location of the CB6 cage molecule. (**D**–**F**) Same as images (**A**–**C**) but images acquired of the rat brain.

We acquired 1 H turbo spin echo (TSE) MR images to correlate the xenon signal with its anatomical location. Immediately prior to ^129^Xe image acquisition, the rat was ventilated with xenon as described above. The imaging sequence began with a saturation pre-pulse consisting of sixteen 20 ms 3-lobe sinc pulses with a 3 sec pulse interval applied at on-resonance (+123.4 ppm) and off-resonance (+260 ppm) chemical shift frequencies. ^129^Xe gradient echo (GE) images of the abdomen and brain were acquired with both off-resonance and on-resonance saturation pre-pulses (Fig. 3A,B,D,E respectively).

**Figure 4:**
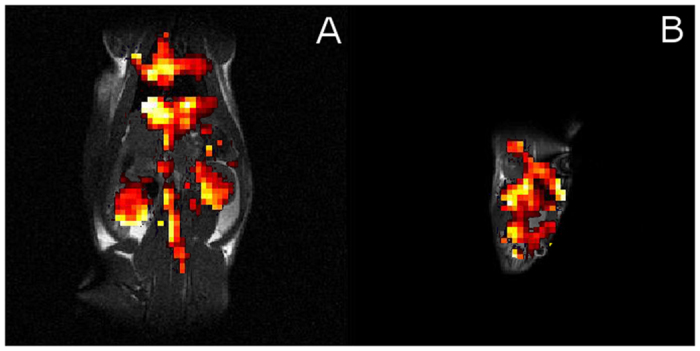
(**A**) HyperCEST saturation map of a rat abdomen zoomed in and overlaid on a 1H MR image showing the location of the CB6 cage contrast agent. Accumulation is noted in the heart (top of image), lungs, liver, aorta (midline), and the kidneys (lateral to aorta, upper abdominal quadrants). (**B**) Same as (**A**) but of the rat brain.

The off-resonance MR images revealed the distribution of xenon throughout the areas of the rat anatomy known to have high perfusion rates such as the brain, aorta, liver, kidney, lungs and heart (Fig. 3A). By applying the HyperCEST saturation pulses we observed a reduction in signal in areas containing the CB6 molecules (Fig. 2B and E). By comparing the on-resonance control images with the off-resonance HyperCEST images (Fig. 2C and F), and registering to the 1 H localizer images, we selectively imaged the areas of CB6 molecules that were localized to the brain, heart, lungs, liver, aorta, and kidneys (Fig. 4). We also created a HyperCEST saturation map in the brain of the rat (Fig. 4B) which demonstrates the localization of CB6 within the brain.

We also conducted a number of control experiments with sham IV injections to ensure that the results that we observed are solely attributable to the location of the cage molecule within the body of the rat. First, we replicated our experiment by injecting 3 mL of PBS buffer containing no CB6 (Supplementary Information Figure S1). We observed an almost identical xenon signal from the brain regardless of off- or on-resonance saturation pre-pulses. A saturation map created from these images yielded almost no HyperCEST signal (Figure S1C). This absence of signal in the absence of a HyperCEST contrast agent provides evidence that the images we document in this report can be attributed to the presence of the CB6 within the body of the rat. We then compared MR images acquired with saturation pre-pulses at an off-resonance frequency and a saturation pre-pulse train at the chemical shift offset of xenon interacting with brain matter (Figure S2). We observed a complete xenon signal depletion (Figure S2B and C) indicating that the HyperCEST effect observed in Fig. 3 can be attributed solely to the HyperCEST effect of the Xe-CB6 interaction and cannot be explained by other processes such as general xenon depolarization or image processing induced artifacts.

## Discussion

In *in vitro* HyperCEST studies, the reduction in the signal of the dissolved phase reservoir of xenon is typically measured. In *in vivo* studies such as these, no such “dissolved pool” exists to measure the HyperCEST depletion; rather than being dissolved in solution, the xenon is dissolved in various tissues and bodily fluids such as lipids in brain matter and blood. It is these pools of xenon interacting with the tissue that constitutes a dissolved phase pool. Xenon atoms diffuse in and out of the CB6 cage, becoming depolarized as they are saturated by the saturation pre-pulse. The saturated xenon atoms, however, continue to interact with various tissues in the body. Despite these additional complexities, our work is an important first proof-of-principle demonstration that HyperCEST imaging is possible in complex biological environments.

We faced a number of challenges to demonstrate the detection of CB6 *in vivo*. In our initial imaging biosensor experiments, we conjugated a cryptophane cage molecule with a PK11195 inflammation detecting affinity tag. We were, however, unable to synthesize sufficient quantities of this imaging biosensor to make *in vivo* experiments feasible^35^. Realizing these limitations, we recently switched our attention to the CB6 supramolecular cage molecule^16^. By using commercially available CB6, which we and others^14^,^16^ determined to exhibit a HyperCEST effect, we were able to inexpensively obtain sufficient quantities of the contrast agent for *in vivo* HP Xe MRI. Second, appropriate radio frequency coils which maximize the form filling factor had to be constructed. We built custom dual-quadrature, dual-tuned (^1^H/^129^Xe) quadrature coils which maximized the filling factor, and therefore the SNR, for rat models. Lastly, a saturation pulse sequence with sufficient specificity for the cage-encapsulated xenon was required. The clinical scanner we used did not originally allow for continuous saturation or a long application of saturation pre-pulse trains (500 ms). We modified the scanner software and MRI pulse sequence to allow for such saturation pre-pulse trains (3 s). Even then, we were initially limited to a short (<50 ms) pulse length, for each individual pulse, which yielded insufficient HyperCEST depletion for imaging purposes. Following additional software modifications, we were able to increase the saturation pulse train length available by the scanner to that which we have described in this work. All of the spectroscopy and imaging experiments described in this study were performed on a clinical 3 T MRI scanner, which bodes well for translation of HP xenon biosensor MRI to clinical applications.

Future advancements will include longer pulse lengths that still respect the specific absorption rate (SAR) limitations for mammalian subjects, which will yield higher depletion and therefore sensitivity. Conjugation of xenon-encapsulating cage molecules with affinity tags to specifically detect pathological molecules has also proven to be difficult, requiring many steps with low yields^21^. Future work will focus on conjugating a cage molecule to an affinity tag with sufficiently high yield to be feasible for detection of disease.

One of the limitations of our study is that we were unable to determine how much of the CB6 actually interacts with xenon and to what extent the CB6 interacts with endogenous tissue. Hydrophobic cage molecules may have a high affinity for the lipid cell surface, resulting in impaired xenon diffusion in and out of the cage cavity^36^. Another limitation of our study is that since our experiments were conducted *in vivo* on a clinical MR scanner, optimized for medical imaging, we were unable to acquire typical depletion spectra that nicely fit exponential Lorenzian curves as described in other works^23^,^30^. Future experiments will focus on optimization of the imaging parameters for *in vivo* MR imaging biosensor studies.

In the future, by combining the MR active cage molecules with an antibody or other affinity tag, a HP xenon MR imaging biosensor could be created to spatially localize areas of pathology within the body. Such biosensors have the potential to provide the imaging based support for personalized medicine. Personalized medicine and precision radiology require specialized imaging modalities that provide imaging biomarkers^37^. These imaging biomarkers may allow for the stratification of patients according to their phenotypic characteristics, which is a requirement of personalized medicine.

With the demonstration of a HP xenon MRI contrast agent *in vivo*, we eagerly anticipate the development and successful *in vivo* demonstration of targeted HP gas MR imaging biosensors that can image localized disease within whole, living organisms.

## Methods

### Sample Preparation

A 10.0 mM solution of cucurbit[6]uril (CB6) was made by dissolving 100 mg of CB6 (Sigma-Aldrich, St. Louis, USA) in 10 mL of 1X phosphate buffered saline (PBS) at pH 7.2. The mixture was heated and stirred for approximately 10 minutes to aid in dissolving the CB6. The solution was allowed to cool to room temperature.

### Rat Preparation

All animal procedures were conducted in accordance with the guidelines set out by the Canadian Council on Animal Care and approved by the Lakehead University Animal Care Committee under animal utilization protocol #1463772. Sprague-Dawley rats (n = 6) (Charles River, Sherbrook QC) weighing between 300–400 g were anesthetized using 3 LPM of isoflurane until their corneal reflex became absent. Once the rats were anaesthetized, a tail vein catheter was placed and an intravenous (IV) infusion of propofol was started (45 mg/kg/hr). A second tail vein catheter (in the other tail vein) was inserted for IV access for CB6 administration.

A midline incision was made in the neck of the rat and the trachea localized. A 1 mm semi-circumscribed incision was made in the trachea, and an endotracheal catheter was inserted into the trachea. The neck was sutured closed. The endotracheal tube was connected to a custom made ventilator^38^ and the rat was placed on oxygen at 60 breaths per minute with a tidal volume of 5 mL.

3 mL of 10 mM CB6 solution was injected over 2 minutes into the tail vein catheter. The rats were placed inside custom dual-tuned (^1^H/^129^Xe) birdcage RF coils. Two 8-rung qual-quadrature, dual-tuned birdcage coils were constructed for rat abdomen and brain imaging, each optimized in size for a maximized filling factor for respective regions of interest. Both coils adopted a four-end ring design to obtain ^129^Xe-1H dual-nuclei imaging ability, facilitating accurate image registration. Following data acquisition, the rats were euthanized by IV injection of pentobarbital.

### Magnetic Resonance Spectroscopy and Imaging

A Philips Achieva 3 T clinical scanner, operating at the ^129^Xe resonance frequency of 35.33 MHz, was used to acquire all MR spectra and MR images. ^129^Xe gas was polarized to 30% using a Xemed polarizer (Xemed, Durham, NH). The magnetic field of the Philips Achieva 3 T scanner was shimmed on the ^1^H signal using a mineral-oil phantom of approximately the same size as the rat to correct for B_0_ inhomogeneities to improve the spectral resolution of the acquisitions. T2-weighted ^1^H turbo spin echo (TSE) multi-slice images (TR = 2 s, TE = 40 ms, flip angle = 12 degrees, slice thickness 2 mm) were acquired from the abdominal and brain regions of the rat with a field of view of 150 mm × 150 mm and a matrix size of 256 × 256, yielding an in-plane resolution of 0.586 mm.

30 minutes following CB6 IV injection, the ventilator was set to dispense xenon to the rat. A 5 second xenon wash in period was provided followed by the continuous administration of an 80% xenon/20% oxygen mixture for the duration of the MR scan. 15 seconds following the beginning of xenon ventilation, a series of FID spectra were acquired with saturation pre-pulses (16 × 20 ms 3-lobe sinc pulses with 3 ms pulse intervals) at various chemical shift offsets. The FID spectra were acquired using a simple pulse-acquire sequence, with a selective “spredrex” excitation pulse of a length of 5 ms and bandwidth of 2849 Hz (80.6 ppm at 3 T)^39^. The receiver bandwidth was 32 kHz (906 ppm at 3 T), which is sufficiently high to prevent xenon gas peak from aliasing back into the acquired spectrum. The data sampling number was 2048, yielding a spectral resolution of 0.44 ppm. HyperCEST depletion was calculated at each chemical shift offset by subtracting the on-resonance HyperCEST SNR from the average of control off-resonance SNR’s (+260 ppm) and divided by the control off-resonance SNR. The HyperCEST depletion values were plotted against the chemical shift offset. Xenon 2D gradient echo images were acquired with a field of view of 150 mm × 150 mm with a matrix size of 64 × 64 and an in-plane resolution of 2.34 mm, slice thickness of 30 mm, TR = 197 ms, TE = 1.67 ms, flip angle of 40 degrees, bandwidth 300 Hz/pixel. On- and off-resonant saturation pre-pulses (+123.4 ppm & +260 ppm respectively) as noted above were applied as part of the acquisition pulse sequence. A wait period of 5 minutes between on- and off-resonant images was provided to ensure that all xenon gas had washed out of the blood stream. For some acquisitions, the images with off-resonant saturation pre-pulses were applied first followed by the images with on-resonant saturation pre-pulses. In other acquisitions the images with on-resonant saturation pre-pulses were applied first. We did this to account for a possible ordering effect to ensure that we were indeed measuring the depolarization of Xe because of the saturation pre-pulses and not the depolarization of xenon with time. Images were analyzed using custom written MATLAB image processing scripts in a method similar to that presented by Klippel *et al*.^23^ and detailed in the Supplementary Information. Briefly, SNR maps were created by dividing each pixel of the MR image by the standard deviation of the noise (see SI). A convolution filter consisting of a 3 × 3 array that approximates a Gaussian distribution with a normalization factor to maintain signal intensity was applied to smooth the images. Saturation maps were produced by the pixel-by-pixel subtraction of the on-resonance image from the off-resonance ^129^Xe images followed by dividing by the off-resonance image. Background noise was removed using a mask consistent with established image processing protocols^23^. Background signal noise outside of the coil was segmented and removed using a mask for Fig. 4, for better image clarity so the underlying localizer MRI of anatomical structures are clearly visible. The HyperCEST saturation maps were overlaid onto the ^1^H images using GIMP image processing software. A complete explanation of the image processing details and the Matlab script is provided in the Supplementary Information.

## Additional Information

**How to cite this article**: Hane, F. T. *et al*. *In vivo* detection of cucurbit[6]uril, a hyperpolarized xenon contrast agent for a xenon magnetic resonance imaging biosensor. *Sci. Rep.* **7**, 41027; doi: 10.1038/srep41027 (2017).

**Publisher's note:** Springer Nature remains neutral with regard to jurisdictional claims in published maps and institutional affiliations.

## Supplementary Material

Supplementary Information
