# Supplementary material for: In vivo detection of cucurbit[6]uril, a hyperpolarized xenon contrast agent for a xenon magnetic resonance imaging biosensor
Source: Sci Rep. 2017 Jan 20;7:41027. doi: 10.1038/srep41027 (PMC5247686; doi:10.1038/srep41027)
Supplement: Supplementary Information [file srep41027-s1.pdf]

In vivo detection of a hyperpolarized xenon magnetic resonance imaging contrast agent

Francis T. Hane [a,b], Tao Li[a], Peter Smylie[a], Raiili M. Pellizzari [a,b], Jennifer A. Plata [a,b], Brenton DeBoef[c], Mitchell S. Albert[*,a,b,d]

[a] Department of Chemistry
Lakehead University
955 Oliver Rd, Thunder Bay ON P7B 5E1 CANADA

[b] Thunder Bay Regional Research institute
980 Oliver Rd, Thunder Bay ON P7B 5E1 CANADA

[c] University of Rhode Island
45 Upper College Rd
Kingston RI 02881 USA

[d] Northern Ontario School of Medicine
955 Oliver Rd, Thunder Bay ON P7B 5E1 CANADA

* Corresponding author: M Albert
E-mail: albertmi@tbh.net

Supplementary Information

Control Images

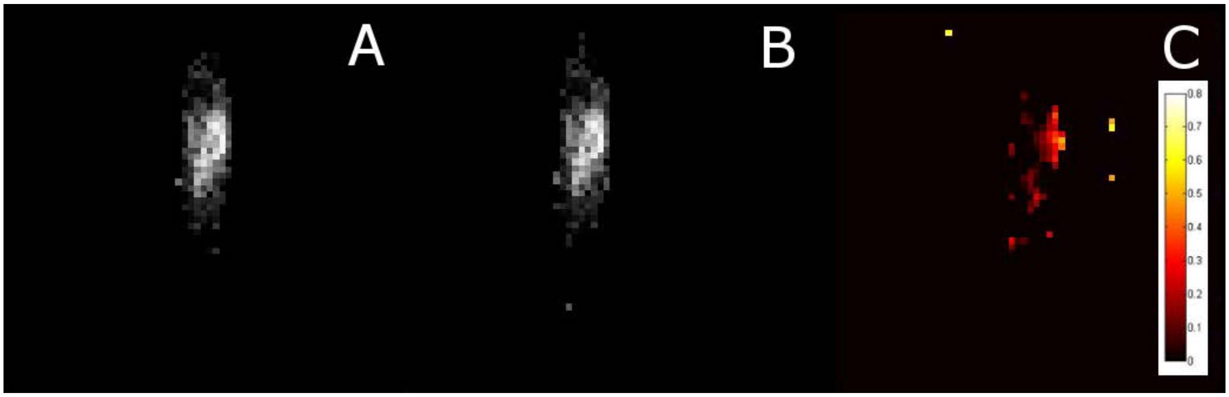

Figure S1. Xe MR control images (NSA=3) of a Sprague-Dawley rat brain absent any cage molecule. A) A 2D gradient echo (GE) Xe MR image of the brain following the application of an off-resonance pre-pulse (+260 ppm). B) Same as A) but following an on-resonance HyperCEST saturation pre-pulse at the chemical shift of the CB6 cage molecule (which was absent) (+123.4). C) A saturation map constructed by subtracting, pixel-by-pixel, the on-resonance HyperCEST image from the off-resonance control image, and dividing by the off-resonance control image as explained in the methods section. Notice the absence of a HyperCEST effect compared to Figures 3 and S2, which is expected because there is no cage molecule present.

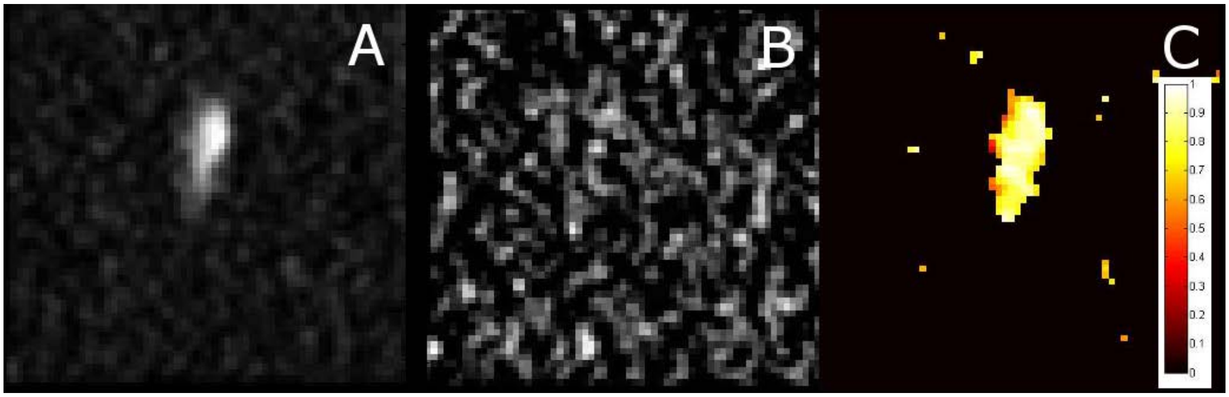

Figure S2. Xe MR control images of a Sprague-Dawley rat brain. A) A 2D gradient echo (GE) Xe MR image of the brain following the application of an off-resonance pre-pulse (+260 ppm). B) Same as A) but following an on-resonance HyperCEST saturation pre-pulse at the chemical shift of xenon dissolved in brain matter (+191.4 ppm). The polarization of the xenon is completely destroyed. C) A saturation map constructed by subtracting, pixel-by-pixel, the on-resonance HyperCEST image from the off-resonance control image, and dividing by the off-resonance control image as explained in the methods section. Compared to Figures 3 and S1 notice a high saturation signal indicating a compete depolarization of the xenon dissolved in brain matter.

Image Post Processing Methodology

<table>
<tr><th>MR Image with Off Resonance Saturation Pulse (Control)</th><th>MR Image with On Resonance Saturation Pulse (HyperCEST)</th></tr>
<tr><td colspan="2">A matrix of the MR image is exported from Philips MR scanner to Matlab for processing.</td></tr>
<tr><td>$$\begin{bmatrix} 0 & 0 & 0 & 0 & 0 & 2 \\ 45 & 21 & 0 & 0 & 35 & 2 \\ 19 & 5 & 0 & 17 & 10 & 5 \\ 17 & 26 & 0 & 0 & 0 & 23 \\ 39 & 21 & 23 & 4 & 0 & 1 \\ 5 & 0 & 2 & 33 & 15 & \dots \end{bmatrix}$$</td><td>$$\begin{bmatrix} 0 & 0 & 0 & 0 & 0 & 0 \\ 20 & 0 & 0 & 26 & 39 & 0 \\ 11 & 1 & 0 & 17 & 29 & 0 \\ 15 & 22 & 6 & 3 & 22 & 16 \\ 31 & 23 & 4 & 0 & 0 & 0 \\ 13 & 7 & 0 & 0 & 0 & \dots \end{bmatrix}$$</td></tr>
<tr><td colspan="2">The matricies are converted to MR images using the Matlab script below.</td></tr>
<tr><td>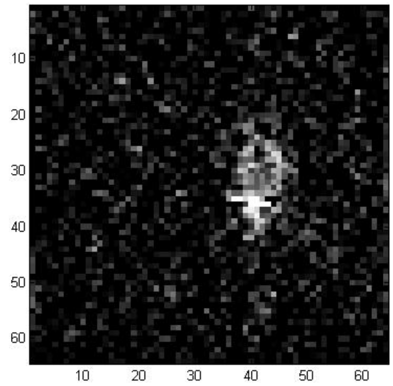</td><td>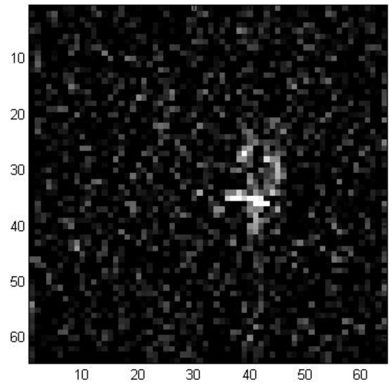</td></tr>
<tr><td colspan="2">The MR images are normalized with respect to noise by sampling a representative noise area, in this case, the first 10x10 voxels. Each pixel is divided by the standard deviation of the noise to obtain an SNR map.</td></tr>
<tr><td>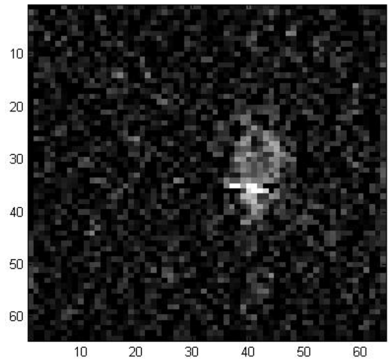</td><td>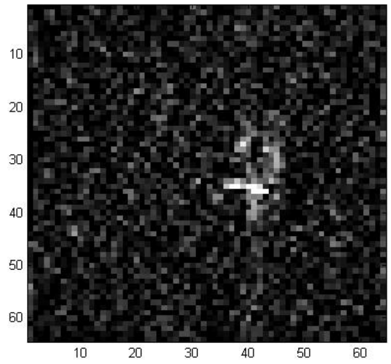</td></tr>
<tr><td colspan="2">The SNR maps are smoothed by convoluting the SNR matrix by a 3x3 Gaussian convolution matrix.</td></tr>
</table>

<table>
<tr><td>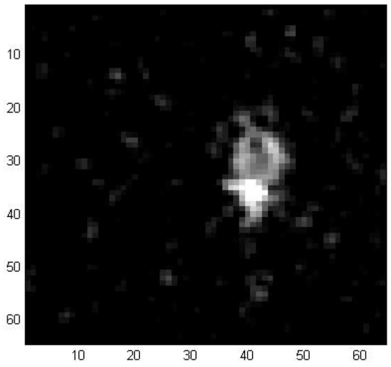</td><td>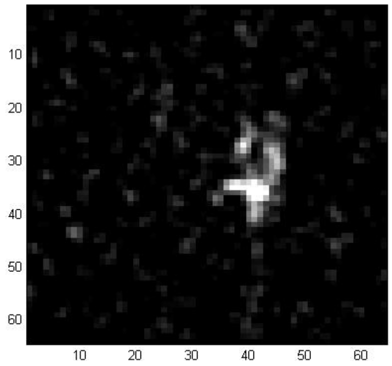</td></tr>
<tr><td colspan="2">A mask of the off-resonance image is taken to remove background noise. This is necessary because during the creation of the HyperCEST saturation map [1]. Masking is necessary because noise subtracted from noise and divided by noise results in an image where the noise is indistinguishable from the object.</td></tr>
<tr><td colspan="2">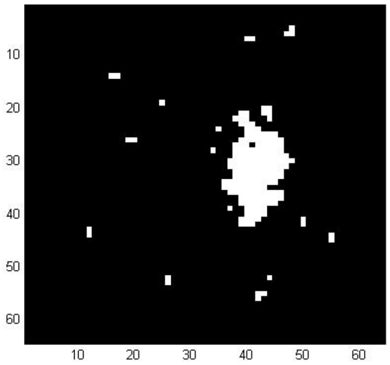</td></tr>
<tr><td colspan="2">The smoothed on Resonance SNR Map is subtracted is subtracted from the off resonance SNR map and divided by the off-resonance SNR map and multiplied by the mask to create a HyperCEST saturation map as seen in figure 3F of the manuscript. The color map is changed to “hot”.</td></tr>
<tr><td colspan="2">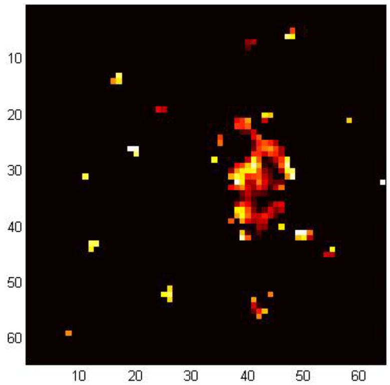</td></tr>
</table>

Table S1

Matlab Image Processing Script

```
% INSTRUCTIONS
% This Script subtracts an on-resonance HyperCEST image from an off-resonance control image and %
divides by the off-resonance image.
% At the first file select box, select the Off-resonance image, at the second file select box, select the on-
resonance image.
%
%% *Sample script for viewing images*

%% *Prepare workspace*
clear all;
close all;
clc;

%% *Select .PAR file*
path = 'C:\Users\Peter\Documents\Philips 3T Data';

%% Select Off-Resonance Image
[filename,pathname] = uigetfile('*.PAR','Select *.PAR file');
OffResonanceparfile = [pathname filename];
[OffResonanceSignal,parms,dims] = GetData_parrec(OffResonanceparfile,'FP','info'); % call function
% which converts MR data to Matlab useable matrix

% Create Off-Resonance SNR map
dataSelected=OffResonanceSignal([1:20],[1:20])
Noise=std(reshape(dataSelected,400,1))
OffResonanceSNRMap = OffResonanceSignal/Noise;

% Select On-Resonance Image
[filename,pathname] = uigetfile('*.PAR','Select *.PAR file');
OnResonanceparfile = [pathname filename];
[OnResonanceSignal,parms,dims] = GetData_parrec(OnResonanceparfile,'FP','info');

% Create On-Resonance SNR map
dataSelected=OnResonanceSignal([1:10],[1:10]);
Noise=std(reshape(dataSelected,100,1));
OnResonanceSNRMap = OnResonanceSignal/Noise;

% Smooth both Images
ConvKern = [1 2 1; 2 4 2; 1 2 1];
SmoothOffResonanceImage = conv2(OffResonanceSNRMap,ConvKern,'same');
SmoothOnResonanceImage = conv2(OnResonanceSNRMap,ConvKern,'same');

% Create mask of Off Resonance Control Image
MaskThreshold=35; % set threshold to select more or less noise
MatrixSize=size(SmoothOffResonanceImage);
mask = zeros(MatrixSize(1),MatrixSize(2));
for i = 1:MatrixSize(1)
  for j = 1:MatrixSize(2)
    if SmoothOffResonanceImage(i,j) < MaskThreshold
      mask(i,j) = 0;
    else mask(i,j) = 1;
    end
```

```
    end
end

% Create a saturation map & Apply Mask
SaturationMap = (SmoothOffResonanceImage-SmoothOnResonanceImage)./SmoothOffResonanceImage;
SaturationMapMasked=SaturationMap.*mask; % Use only data from within mask
imagesc(SaturationMapMasked);

% apply image parameters
colormap hot;
axis square;
caxis([0 0.8]);
colorbar;
```

References

1. Witte, C. *et al.* Live-cell MRI with Xenon Hyper-CEST Biosensors Targeted to Metabolically Labeled Cell-Surface Glycans. *Angew. Chemie (International Ed.* **54,** 2806–2810 (2015).
